# Responsive Neurostimulation Therapy Outcomes in Two Small Bilateral Thalamic and Corticothalamic Subgroups of Patients with Drug-Resistant Epilepsy—A Single-Center Experience

**DOI:** 10.3390/neurosci7040092

**Published:** 2026-08-21

**Authors:** Ramya Krothapally, Ghazala Perven, Divya Veerapaneni, Irina Podkorytova

**Affiliations:** Department of Neurology, University of Texas Southwestern Medical Center, Dallas, TX 75390, USA; ramya.krothapally@utsouthwestern.edu (R.K.); ghazala.perven@utsouthwestern.edu (G.P.); divya.veerapaneni@utsouthwestern.edu (D.V.)

**Keywords:** anterior nucleus of thalamus, centromedian nucleus of thalamus, corticothalamic, responsive neurostimulation, RNS, pulvinar, thalamic neuromodulation

## Abstract

Responsive neurostimulation (RNS) is a neuromodulation option for treatment of drug-resistant epilepsy (DRE). There is limited data reviewing bilateral thalamic and corticothalamic RNS therapy response. We performed a retrospective analysis of 21 patients undergoing RNS implantation with either bilateral thalamic (*n* = 17) or corticothalamic (*n* = 4) leads and ≥6 months of follow-up. Patients were classified as responders (≥50% seizure frequency reduction) vs. non-responders (<50% seizure frequency reduction). Among the bilateral thalamic cohort, 12/17 (70.6%) patients were responders, and 15/17 (88.2%) patients reported reduction in seizure severity. Pre-RNS seizure frequency was higher in bilateral thalamic nonresponders, although not found to be significant (median 13 vs. 30 seizures per month, *p* = 0.20). Among the corticothalamic cohort, 3/4 (75.0%) patients were responders, and all patients demonstrated improvement in seizure severity. These findings suggest that although bilateral thalamic targets were selected at our center more often, both bilateral thalamic and corticothalamic RNS approaches provided seizure frequency reduction. The majority of patients, including non-responders, reported seizure severity alleviation.

## 1. Introduction

Drug-resistant epilepsy (DRE) affects up to one-third of epilepsy patients, of which certain individuals may not be candidates for resective or ablative surgery due to multifocal involvement or seizure onset zones within the eloquent cortex [1]. Responsive neurostimulation (RNS; NeuroPace, Inc., Mountain View, CA, USA) is a closed-loop neuromodulation therapy that delivers stimulation in response to detected epileptiform activity, shown to achieve sustained reduction in seizure frequency and severity for DRE patients unable to undergo resection [2,3].

The thalamus has been identified as a neuromodulation target, reflecting a shift toward understanding epilepsy as a network disorder rather than a single focal pathology, and it functions as a central structure facilitating seizure propagation and synchronization across cortical regions [4,5,6]. Stimulation of specific nuclei—including the anterior (ANT), centromedian (CMT), and pulvinar—targets distinct but overlapping epileptic networks, with the ANT implicated in limbic circuits, the CMT in frontal, generalized, and multifocal networks, and the pulvinar in posterior and temporoparietal propagation pathways [7,8,9]. Building on this network-based concept, corticothalamic approaches have been used to simultaneously target cortical seizure onset zones and ipsilateral thalamic nuclei, enabling modulation at multiple nodes within the epileptic circuit [10,11]. Neuromodulation of these circuits is thought to disrupt pathological synchrony and promote network-level reorganization, with unilateral or bilateral thalamic RNS [12]. It remains unclear whether corticothalamic configurations provide additional benefit over thalamic-only approaches.

In this study, we review the clinical outcomes of bilateral thalamic and corticothalamic RNS configurations in patients with DRE in a single center to contribute to the understanding of the role of network-level targeting in neuromodulation therapy between responders and nonresponders.

## 2. Materials and Methods

### 2.1. Patients

**Figure 1:**
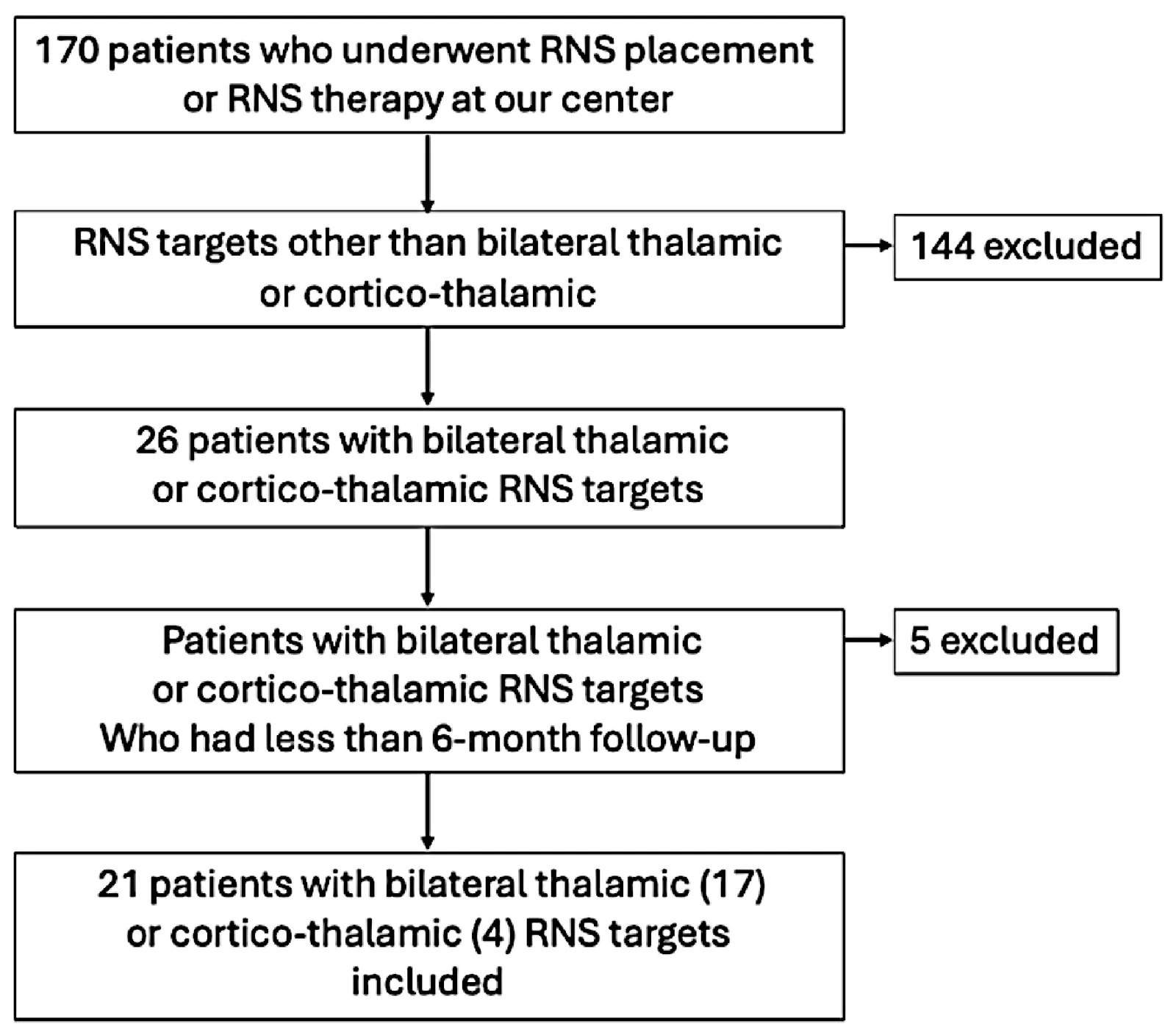
Patient-selection flow diagram.

This is a retrospective case series approved by the Institutional Review Board. We reviewed 170 consecutive patients who underwent RNS placement or RNS therapy at our center from 2014 to 2025 and found 21 patients who met the following inclusion criteria: (1) RNS targeting bilateral thalamic nuclei or corticothalamic RNS targets with all potential thalamic nuclei sampling, including ANT, CMT, and pulvinar, (2) had ≥ 6 months of post RNS implantation follow-up, and (3) ≥ 18 years of age at time of the most recent follow-up RNS clinic visit at our center. 144 out of 170 RNS patients had non-thalamic RNS targets and were excluded from this study. An additional 5 patients (4 with bilateral CMT leads and one with left amygdala and left ANT leads) who had less than 6-month follow-up duration with RNS therapy were excluded from this study. A patient-selection flow diagram is depicted in Figure 1.

Baseline patient characteristics were collected, such as age of epilepsy onset, age of RNS implantation, prior neurosurgical interventions, results of genetic testing, magnetic resonance imaging (MRI) findings, scalp EEG and stereoelectroencephalography (SEEG) evaluation results, and RNS implantation sites.

### 2.2. Lead Placement Decision Making

Recommendations for lead placement were determined during a multidisciplinary surgical conference dedicated to reviewing the presurgical and intracranial SEEG data, if available. The decision for nuclei selection was dependent on existing knowledge of thalamic connections and the determined onset of seizures. Leads in the corticothalamic group were placed ipsilateral to the identified area of epileptogenesis.

### 2.3. Patient Outcomes

The baseline seizure frequency prior to RNS was established based on patient reports at the last clinic visit prior to RNS implantation. Primary seizure outcomes were defined as ≥50% seizure reduction as responders and/or reduction in seizure severity compared with pre-implantation baseline. Seizure outcomes at the most recent post-RNS implantation follow-up were determined based on the patient reports obtained from the electronic medical records. Seizure severity reduction was defined as decreased seizure duration, decrease in motor manifestations, and/or decrease in postictal symptoms per patient report.

### 2.4. Statistical Analysis

Statistical analysis was performed using R 4.3.3 (R Core Team, Vienna, Austria, 2024) and RStudio 2023.12.1.402 (Posit Software, PBC, Boston, MA, USA). The Wilcoxon rank sum test was used to compare seizure frequency and percent decrease in seizure frequency between bilateral thalamic patient responders and nonresponders due to small sample size. Statistical analysis between corticothalamic responders and nonresponders was not performed given that the nonresponder group comprised one patient.

## 3. Results

### 3.1. Baseline Patient Characteristics

Individual patient characteristics are described in Table 1. Twenty-one patients underwent RNS implantation with either bilateral thalamic (BT) leads (*n* = 17, ten CMT and seven ANT) with 12 responders (seven CMT, and three had generalized epilepsy) and five nonresponders (three CMT, one had generalized epilepsy, one ANT, and BT ANT leads were replaced with BT CMT in one patient), or corticothalamic (CT) configurations (*n* = 4, one CMT, two ANT, and one pulvinar) with three responders (one ANT, CMT, and pulvinar, respectively) and one nonresponder (left ANT was replaced with right CMT).

The median age of epilepsy onset was similar for BT responders and nonresponders at 6 years (quartile 1 to quartile 3 (Q1–Q3) 2–10) and 5 years (Q1–Q3 2–7), respectively, and for the CT group, it was 15 years (range 15–16) for responders and 24 years for the CT nonresponder. The median age of RNS implantation was 29 years (Q1–Q3 24–44) for BT responders, 23 years (Q1–Q3 19–27) for BT nonresponders, 33 years (Q1–Q3 31–40) for CT responders, and 56 years for the CT nonresponder.

### 3.2. Etiologies

Among bilateral thalamic responders, 9/12 (75.0%) patients had an unknown etiology and 3/12 (25.0%) had a structural etiology. Among bilateral thalamic nonresponders, 3/5 (60.0%) patients had unknown, 1/5 genetic (20.0%), and 1/5 infectious (20.0%) etiologies. Etiologies for corticothalamic responders included unknown and structural (1/3, 33.3% and 2/3, 66.7%, respectively), while the single corticothalamic nonresponder had infectious epilepsy (1/1, 100%).

### 3.3. Prior Surgical Therapies

Nine out of 12 (75%) of bilateral thalamic responders had prior neurosurgical interventions, including 5/9 (55.6%) patients with vagus nerve stimulation (VNS, VNS Therapy^®^, LivaNova, Houston, TX, USA) placement alone, 2/9 (22.2%) resection, and 2/9 (22.2%) resection or laser interstitial thermal therapy (LITT, Monteris Medical, Minnetonka, MN, USA) and VNS. In total, 4/5 (80.0%) of the bilateral thalamic nonresponders had prior surgical intervention, including 2/4 (50.0%) patients with VNS placement alone, 1/4 (25.0%) resection, and 1/4 (25.0%) resection plus VNS placement. In total, 1/3 (33.3%) of corticothalamic responders had prior intervention. The patient with left frontal encephalomalacia had left frontal resection post-SEEG. One non-responder from the corticothalamic cohort has not had therapeutic neurosurgery before RNS placement.

### 3.4. Imaging Findings

Six out of 12 (50%) bilateral thalamic responders had MRI lesions that could be epileptogenic, but SEEG confirmed the ictal onset from the lesion only in two out of six patients: patient 5 with bilateral subcortical band heterotopia, and patient 6 with right temporal lobe encephalomalacia (one of the multiple SEEG ictal onsets). In two out of six patients, the ictal SEEG onsets were discordant with brain MRI lesion location (patients 8 and 9 with bilateral occipital encephalomalacia and left temporal lobe volume loss, respectively). Patients 2 and 4 with bifrontal T2 hyperintensities and multiple cortico-subcortical encephalomalacia areas, respectively, have not had invasive EEG evaluations.

In total, 1/5 (20%) bilateral thalamic nonresponders had a possible epileptogenic MRI lesion; patient 14 had right frontal T2 hyperintensity on brain MRI, but the SEEG ictal onset was broad bilateral frontal involving bilateral insulae. In total, 1/3 (33.3%) of corticothalamic responders and the corticothalamic nonresponder had lesional brain MRI partially concordant with SEEG ictal onsets. Individual imaging findings are reported in Table 1.

### 3.5. Invasive EEG Evaluation

Eight out of 12 (66.7%) of bilateral thalamic responders underwent SEEG evaluation, with 3/8 (37.5%) patients experiencing a broad ictal onset and 5/8 (62.5%) patients having multifocal ictal onsets. In total, 3/5 (60%) of bilateral thalamic nonresponders underwent SEEG evaluation, with 1/3 (33.3%) having a broad ictal onset and 2/3 (66.7%) with multifocal ictal onsets. All corticothalamic patients underwent SEEG evaluation, with 3/3 corticothalamic responders experiencing a broad ictal onset and the corticothalamic nonresponder having multifocal ictal onsets.

### 3.6. RNS Targeting

Among the cohort, 9/21 (42.9%) patients had ANT implantation, 11/21 (52.4%) CMT implantation, and 1/21 (4.7%) pulvinar implantation. In total, 6/9 (66.7%) patients with ANT implantation (five bilateral thalamic), 8/11 (72.7%) patients with CMT implantation (seven bilateral thalamic), and 1/1 patient with corticothalamic leads targeting pulvinar implantation were responders (Table 2). The patient from the corticothalamic group who had right parietal and right pulvinar RNS lead placement had right parietal resection post-SEEG and RNS lead placement as one procedure.

The following clinical and neurophysiological features favored ANT, CMT, or pulvinar implantation: 1. early cases implanted before the centromedian nucleus of the thalamus became a standard RNS target, with six patients (four responders); 2. ANT target for temporal lobe epilepsy based on Stimulation of the Anterior Nucleus of the Thalamus for Epilepsy (SANTE) trial results, and mesiotemporal lobe epilepsy or epilepsies involving limbic circuits, with three patients (two responders); 3. CMT target for generalized epilepsy, with four patients (three responders); 4. CMT target for frontal onset focal epilepsy, with one patient (responder); 5. CMT target for diffuse onset regional epilepsy, with six patients (four responders); 6. pulvinar nucleus target for posterior quadrant epilepsy, with one patient (responder)—see Table 1. Supplementary Figure S1 demonstrates examples of the RNS leads location in the thalamus.

Three out of 21 (14.3%) patients underwent RNS lead switching; two patients underwent replacement of RNS thalamic targets from ANT to CMT (one bilateral thalamic and one corticothalamic), and one patient from the bilateral thalamic group switched from ANT to bilateral hippocampal leads due to reported poor seizure control after initial implantation. The two patients with leads switched to CMT remained nonresponders, while the patient with subsequent hippocampal implantation was a responder with bilateral ANT and then with bilateral hippocampal RNS therapy. Detailed outcomes after additional interventions are described under Section 3.8.

### 3.7. Seizure Characteristics and Clinical Outcomes

Overall cohort response rate was 15/21 (71.4%), achieving ≥50% seizure reduction, with subgroup rates at 12/17 (70.6%) bilateral thalamic patients; seven CMT and 3/4 (75.0%) corticothalamic patients; and one ANT, CMT and pulvinar, respectively. Among the nonresponders, 5/6 (83.3%) reported reduction in seizure severity despite not having ≥50% seizure reduction. The median pre-RNS seizure frequency per month for bilateral thalamic responders was 13 seizures (Q1–Q3 6–45), which was lower than the seizure frequency for bilateral thalamic nonresponders at 30 seizures (Q1–Q3 30–75), although not found to be significant (Wilcoxon Rank Sum test, *p* = 0.20).

The median pre-RNS seizure frequency per month for the corticothalamic group was eight seizures (Q1–Q3 7–11) for the responders and 75 seizures for the nonresponder.

**Figure 2:**
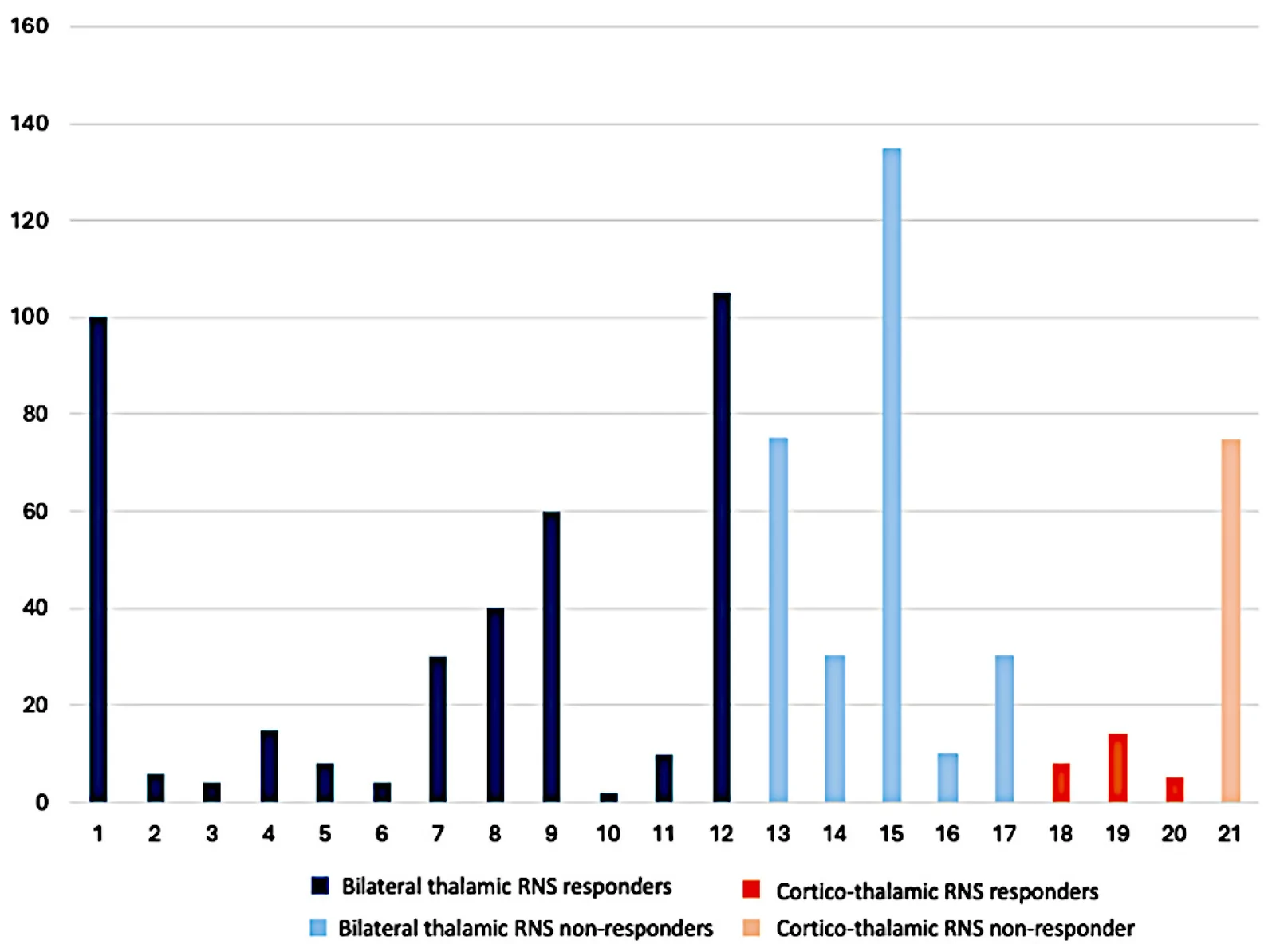
Patient-reported pre-RNS seizure frequency per month: x, patient ID, y, seizure frequency per month.

Figure 2 depicts patient-reported pre-RNS seizure frequency per month.

Of the seizures reported, 11/17 (64.7%) patients in the bilateral thalamic group had focal to bilateral tonic–clonic (FBTC) or generalized tonic–clonic (GTC) with a median yearly pre-RNS frequency of six seizures (Q1–Q3 4–28) among nine responders and 125 seizures among two nonresponders. In total, 5/11 patients continued to experience FBTC/GTC post-RNS, with four responders with a median yearly frequency of 1 (Q1–Q3 1–8), and one nonresponder with a median frequency of 125 seizures. The bilateral thalamic nonresponder did report a reduction in the severity of seizures, including FBTC/GTC seizures, of 10%. In total, 3/4 (75%) patients in the corticothalamic group reported FBTC/GTC seizures prior to RNS with a median frequency of four seizures, and all patients reported resolution of FBTCs after RNS implantation.

The median percent of overall post-RNS seizure frequency reduction was 75% (Q1–Q3 74–81%) for bilateral thalamic responders compared to 30% (Q1–Q3 10–40%) for the bilateral thalamic nonresponders. The median percent of seizure reduction post RNS for the corticothalamic group was 100% (Q1–Q3 95–100%) for the responders and 40% for the nonresponders (Table 2). RNS therapy outcomes at common intervals are depicted in Table 3. Table 4 demonstrates anti-seizure medications pre-RNS and at the most recent RNS follow-up visit.

### 3.8. Outcomes After Additional Interventions

Favorable seizure outcome in patient 19 who underwent right parietal cortex resection along with right parietal and right pulvinar RNS lead placement as one procedure post-SEEG could be entirely or partially related to the effect of the resective surgery and ASM but not RNS therapy alone.

Three patients underwent RNS lead replacement, changing the RNS therapy target. Two out of three patients were non-responders to RNS therapy before and after the RNS target change, and one out of three patients was a responder to RNS therapy before and after the RNS target change.

Patient 16 was a non-responder to the RNS therapy before and after the RNS target change. This patient had a positive CHRN4A gene mutation and seizure onset at 1 year of age. He underwent two SEEG evaluations at an external hospital. The first SEEG showed a broad right hemispheric ictal onset with possible right mesial temporal initial leading. The patient underwent right temporal lobectomy, but his seizures did not improve after resective surgery. The patient underwent the second SEEG evaluation at the outside hospital, which showed a broad left temporal and left insula ictal onset. Subsequently, the patient was discussed for epilepsy surgery at our center, and bilateral ANT RNS lead placement was recommended. He was our first thalamic RNS case implanted before the centromedian nucleus of the thalamus became a standard RNS target. Due to ineffectiveness with ANT RNS therapy for 69 months, the RNS leads were explanted from bilateral ANT and placed to CMT bilaterally. The patient continues to be a non-responder to RNS therapy with less than 50% seizure frequency reduction at 12-month follow-up with bilateral CMT RNS.

Patient 21 had left temporal lobe encephalomalacia and DRE after herpes virus encephalitis. The bilateral SEEG evaluation localized multifocal ictal onsets within the left temporal neocortex and left parietal cortex and documented non-epileptic events. The patient was a non-responder to left ANT and left temporal neocortical RNS therapy for 13 months per patient-reported seizures. The left ANT lead was replaced with a CMT lead based on ineffectiveness and patient-reported seizures suggesting right frontal lobe ictal onset based on seizure semiology, which were not detected with left ANT and left RNS leads. At 34-month follow-up with the right CMT and left temporal neocortical RNS therapy, the patient continues reporting seizure frequency with less than 50% reduction compared to pre-RNS placement baseline. The RNS detects multiple per-month magnet swipes without correlation with the ictal RNS ECOGs. All recorded RNS seizures were seen over the left temporal neocortical lead with frequency of none or several per year (no seizures in 2026, five seizures in 2025, seven seizures in 2024, etc.). Given the multifocal nature of the patient’s post-encephalitis epilepsy, two RNS leads could miss the ictal onsets. However, given documented history of non-epileptic events, the patient-reported seizures could be non-epileptic.

Patient 12 had a remote history of head trauma with skull fracture in infancy and DRE onset decades later. The brain MRI was non-lesional, SEEG localized multifocal seizures within the right parietal, right mesial and lateral temporal, and left mesial temporal structures. Additionally, non-epileptic events were captured and reported as typical patients’ seizures. She was a responder to bilateral RNS ANT therapy with more than 50% seizure frequency reduction at 23-month follow-up. The decision to remove bilateral ANT RNS leads and place bilateral hippocampal RNS leads was based on the presence of left ANT lead migration to the ventricle and patient-reported seizures that were not captured with bilateral ANT RNS leads. The patient continues to respond to RNS therapy, reporting more than 50% seizure frequency reduction. We may hypothesize that two ANT RNS leads might miss “small” seizures which do not spread to the thalamus. However, given documented history of non-epileptic events, the patient-reported seizures could be non-epileptic.

### 3.9. Surgical Complications

Patients 4 and 19 had wound infection at the location of the RNS pulse generator. Patient 4 underwent wound/battery site revision with the RNS pulse generator explant 2 months after the initial RNS placement, followed by the RNS battery re-implant 8 months after removal. Patient 19 underwent wound/battery site revision with the RNS pulse generator replacement 2 months after the initial RNS placement. Patient 12 had left ANT lead migration to the paramedian third ventricle diagnosed with images 13 months after the initial RNS placement. The bilateral ANT RNS leads were removed, and bilateral hippocampal RNS leads were placed (detailed description in Section 3.8).

No other complications related to RNS placement or RNS therapy were observed.

## 4. Discussion

Our study of two small clinical subgroups of patients with bilateral thalamic and corticothalamic RNS leads demonstrates that physicians selected bilateral thalamic targets for RNS therapy more often compared to the corticothalamic targets (81% vs. 19%, respectively) in our cohort of 21 patients. Overall, both the bilateral thalamic and corticothalamic subgroups had a high number of responders to RNS therapy, with ≥50% seizure frequency reduction at 70.6% and 75.0%, respectively. Fifteen out of 17 (88.2%) patients in the bilateral thalamic subgroup and all patients with corticothalamic leads reported reduction in seizure severity. In addition, five out of nine bilateral thalamic responders, one out of two bilateral thalamic non-responders, and all four patients from the corticothalamic subgroup who had FBTC or GTC pre-RNS reported resolution of convulsive seizures with RNS therapy. These outcomes suggest that both bilateral thalamic and corticothalamic approaches can achieve seizure-burden reduction.

Our outcomes are similar to previous cohort studies reporting seizure outcomes, defined as ≥50% of seizure frequency reduction, for both unilateral and bilateral thalamic neuromodulation between 60 and 79%, and additional small case series reporting higher percentages than this [10,13,14,15,16,17]. The majority of patients with ANT and CMT sampling were found to be responders in this study. Our patients with idiopathic generalized epilepsy (IGE) were implanted with bilateral CMT thalamic leads, with three out of four (75%) being responders reporting ≥50% reduction in overall seizure frequency, including GTCSs, of which two patients had complete resolution of GTCs at most recent follow-up, which supports the recommendation for CMT for IGE [18,19]. While bilateral thalamic implantation was more common among our cohort, likely due to a wider range of implications for multifocal and generalized epilepsies as well as earlier adoption of bilateral thalamic RNS implantation strategy, our data adds to growing support for corticothalamic closed loop treatment of DRE given favorable outcomes among our small subgroup [7,16].

There is limited data on patients with pulvinar nucleus implantation compared to ANT and CMT nuclei, although initial review of this strategy suggests that it is well tolerated and can achieve significant seizure reduction when implanted in posterior quadrant epilepsies [20]. Burdette et al. described three patients with corticothalamic leads in the pulvinar nucleus and either occipital or parietal cortex, with all patients having >50% seizure reduction [21]. In our cohort, we identified one patient implanted with one lead in the pulvinar nucleus and the other lead in the right parietal cortex, in addition to a right parietal resection of a non-eloquent seizure onset zone after SEEG, which demonstrated a broad ictal onset over the right precuneus, parietal, and angular gyrus and was found to be a responder with full resolution of all seizures. Such a favorable outcome might be related to the effect of the resective surgery and ASM but not RNS therapy alone.

Notably, in the bilateral thalamic cohort, nonresponders had a higher overall rate of seizures prior to RNS implantation compared to responders, although this was not found to be statistically significant. This trend was also seen with the FBTC/GTC semiology, although the sample size is small and only observations can be made. A difference in seizure rates between responders and nonresponders as a potential indicator for poor response to RNS has not been robustly reported before. In contrast, one study by Hu et al. suggests low seizure burden as a possible risk factor for having <50% seizure frequency reduction, likely due to limited area for improvement, and additional large-scale RNS studies have not identified any specific clinical features to suggest a patient might be a nonresponder [22,23]. As seen with other RNS configurations, RNS thalamic neuromodulation progressively improves seizure frequency over years as demonstrated by a study by Fields et al., reporting median seizure reduction of 33% at 6 months, 55% at 1 year, 65% at 2 years, and 74% at >2 years, although majority of the nonresponders in our cohort had longer than 1 year follow-up at the time of this study suggesting that this was not a contributing factor [15]. The reason for this trend within our cohort is unclear and may be an area of further interest in future thalamic neuromodulation studies.

Our study did show that the percentage of seizure frequency reduction among responders was significantly higher compared to the percentage of seizure reduction among nonresponders in the bilateral thalamic group. Huynh et al. also reported similar findings among patients diagnosed with DRE secondary to viral brain infection implanted with RNS who were noted to be responders with >95% seizure reduction in comparison to nonresponders with a low average seizure reduction of 14% [24]. The lack of robust response in certain patients may be multifactorial and may relate to the higher seizure burden that was noted in our study, although our cohort was too small to corroborate this. There are several additional identified factors in the literature that have been suggested to influence outcomes in thalamic neuromodulation, which could be considered. Roa et al. report an observation that having an extratemporal seizure onset zone (SOZ) was less favorable than a temporal SOZ in a cohort of 23 patients implanted with ANT or CMT targets [14]. We note that the majority of our patients had extratemporal SOZ regardless of seizure outcomes. Imaging tractography has been shown to identify degrees of thalamocortical connectivity, with high connectivity being associated with better outcomes [25,26,27]. While this was not specifically completed for our patients, it could be a tool used in the future to better tailor RNS lead implantation.

We observed that responders had a higher number of lesional MRIs compared to nonresponders among the bilateral thalamic subgroup (6/12, 50% vs. 1/5, 20%). Study by Jobst et al. found a significantly higher seizure reduction in patients with a lesional MRI treated with RNS compared to patients with normal MRI, although both groups of patients reported improvement with neuromodulation [28]. Additional clinical factors were not found to be notably different between responders and nonresponders, including prior neurosurgical interventions, type or etiology of epilepsy, and identified SEEG ictal onset. While the majority of our patients with focal epilepsy underwent SEEG monitoring, we did not report whether thalamic sampling was completed and its role in guiding neuromodulation targeting in this study. However, this practice is becoming more supported as safe and better, allowing for targeted treatment for individual patients [29,30]. There has not been a significant difference in response to RNS in focal compared to multifocal epilepsies in the prior large cohort studies [23,31].

In two out of 3 patients who underwent RNS leads switching, one of the reasons of this operation was that the patient-reported seizures were not captured by the RNS leads. We may hypothesize that the thalamic leads might miss “small” seizures which do not spread to the thalamus. However, both these patients had documented history of non-epileptic events, therefore the patient-reported seizures which were not captured by the RNS leads might be non-epileptic.

Several studies reported the high-frequency oscillations and fast ripples as emerging network biomarkers that may eventually help refine detection and RNS target selection [32,33].

This study is limited by its small sample size, particularly within the corticothalamic subgroup, which restricts statistical power and generalizability. The retrospective design introduces potential selection bias, and heterogeneity in seizure types, etiologies, medication, and targeting strategies, which may confound interpretation. Additionally, seizure frequency and seizure severity reduction data at each interval follow-up after RNS placement were derived from clinical documentation and patient reports, introducing potential reporting variability in the interpretation of response to RNS. Finally, detailed longitudinal changes in seizure semiology and exact RNS detection settings and stimulation parameters were not reviewed, limiting more detailed analysis. Settings such as stimulation frequency, pulse width, current intensity, and detection thresholds have a direct impact on therapeutic efficacy. Without these data, it is impossible to distinguish whether outcomes reflect target selection or parameter optimization.

## 5. Conclusions

This study shows comparable outcomes with bilateral thalamic and corticothalamic implantation strategies in two small clinical subgroups, supporting the growing evidence for network-guided neuromodulation arrangement in DRE. Nonresponders in our cohort had a higher seizure burden and fewer lesional brain MRIs, which might portend less favorable response to thalamic RNS, although this was not statistically proven. These findings represent descriptive clinical experience rather than comparative effectiveness or patient-selection markers. Future studies incorporating prospective design, larger cohorts, and integration of advanced network mapping techniques will be essential to refine patient selection and optimize therapeutic response.

## Figures and Tables

**Table 1:** Patient characteristics, surgical procedures, seizure frequency, EEG data, thalamic targets, and RNS therapy outcomes.

Patient ID	Sex, Handedness, Age at Seizure Onset, Age at RNS Placement (yrs)	Prior Resection or LITT (Y/N, Location); Surgical Pathology	Prior VNS; VNS Therapy Received Pre-RNS; VNS Therapy Received at Last f/u (Y/N)	Hx of GTC/FBTC (Y/N); >1/yr Pre-RNS, (Y/N), Frequency/yr	Average Seizure Frequency Pre-RNS, All Seizures/mo	Presumed Epilepsy Etiology	Scalp EEG Onset of Habitual Seizures	SEEG Onset of Habitual Seizures (If Completed)	Initial MRI Findings Prior to Implantation	RNS Targets, Target Selection *	≥50% Seizure Reduction; Seizure Severity Reduction (Y/N); f/u Duration (mo)	Presence of GTC/FBTC with RNS (Y/N); Average Frequency/yr	% Decrease in All Seizure Types; RNS Programming Visits, *n*
Bilateral thalamic leads													
1	Fe, R, 0, 29	N; N/A; N/A	Y; Y; Y	Y; Y, 300	100	U	F; nonlat/nonloc	N/A	Unremarkable	Bi CMT ^5^	Y; Y; 67	Y; 30	75%; 15
2	M, R, 4, 16	N; N/A; N/A	N; N/A, N/A	Y; Y, 6	6	U	G	N/A	BiF subcort T2 punctate hyperintensities	Bi CMT ^3^	Y; Y; 49	Y; 1	75%; 3
3	M, R, 2, 51	Y, R ATL; not available, OSH	N; N/A; N/A	Y; Y, 2	4	U	G	broad Bi FPT	Mild chronic MV changes	Bi CMT ^5^	Y; N; 29	Y; 1	75%; 8
4	M, R, 1, 22	N; N/A; N/A	Y; Y; Y	Y; Y, 75	15	S	G	N/A	Multiple cort-subcort encephalomalacia	Bi CMT ^3^	Y; Y; 25	N; N/A	75%; 7
5	Fe, R, 8, 25	Y, BiP LITT; N/A	Y; N; N	Y; Y, 12	8	S	F; nonlat/nonloc	MF; L/RP cortex, broad BiPF	Extensive subcort band heterotopia	Bi CMT ^5^	Y; Y; 21	N; N/A	60%; 7
6	M, R, 2, 41	N; N/A; N/A	Y; Y; Y	Y, N, N/A	4	U	F; BiO	MF; L ant insula/AC/OFC, L post insula, RT operculum/STG	Possible R ITG lesion, R STG cystic encephalomalacia	Bi CMT ^5^	Y; Y; 15	N; N/A	60%; 7
7	Fe, R, 13, 25	N; N/A; N/A	Y; Y; Y	Y; Y, 6	30	U	G	N/A	Unremarkable	Bi CMT ^3^	Y; Y; 14	N; N/A	100%; 4
8	M, R, 0, 21	N; N/A; N/A	Y; Y; Y	Y; Y, 2	40	S	MF; L vertex, RF	MF; RF, LF, BiP	BiO encephalomalacia	Bi ANT ^1^	Y; Y; 76	N; N/A	75%; 14
9	M, R, 7, 29	Y, LF lobectomy; mild MCD, OSH	Y; Y; Y	N, N, N/A	60	U	N/A	MF; L/R insula/operculum	Possible LT volume loss	Bi ANT ^1^	Y; Y; 55	N; N/A	100%; 12
10	M, R, 25, 31	Y, R SFG/AC resection; Normal	N; N/A; N/A	N, N, N/A	2	U	F; LT	broad R SFG/RT	Edema of R amygdala	Bi ANT ^1^	Y; Y; 49	N; N/A	100%; 10
11	Fe, R, 13, 52	N; N/A; N/A	N; N/A; N/A	N, N, N/A	10	U	F; broad BiFT	broad B FT	R HC T2 hyperintensity	Bi ANT ^1^	Y; Y; 41	N; N/A	70%; 14
12	Fe, R, 9, 51	N; N/A; N/A	N; N/A, None	Y; Y, 4	105	U	F; R FT	MF; ant RT, R angular gyrus, L HC	Unremarkable	Bi ANT ^2^ then Bi HC	Y; Y; 23	Y; 1	75%; 7
13	M, R, 7, 27	N; N/A; N/A	Y; N; N	N, N, N/A	75	U	G	N/A	Unremarkable	Bi CMT ^3^	N; Y; 47	N; N/A	40%; 14
14	Fe, R, 2, 23	Y, R insular/frontal operculum/LF pole resection; normal	N; N/A; N/A	N, N, N/A	30	I	F; nonlat/nonloc	broad BiF/ant insula R > L	RF T2 hyperintensity	Bi CMT ^5^	N; N; 15	N; N/A	0%; 5
15	Fe, R, 17, 19	N; N/A; N/A	N; N/A; N/A	Y; Y, 125	135	U	F; LT, nonlat	MF; L OFC, L TP	Incomplete L HC inversion	Bi CMT ^5^	N; Y; 15	Y; 125	10%; 7
16	M, R, 1, 19	Y, R ATL; FCD 2A, OSH	Y; Y; Y	Y; Y, 125	10	Gen	MF; OSH SEEG11—R hemisphere, OSH SEEG2—broad LT, L insula	N/A	Unremarkable	BiANT ^1^ then BiCMT ^5^	N; Y; 81	N; N/A	40%; 30
17	M, R, 5, 41	N; N/A; N/A	Y; Y; Y	N, N, N/A	30	U	F; nonlat/nonloc	MF; L OFC, L MFG, BiF	Unremarkable	Bi ANT ^5^	N; Y; 60	N; N/A	30%; 18
Corticothalamic leads													
18	M, R, 17, 47	N; N/A; N/A	N; N/A; N/A	Y; Y, 6	8	S	F; nonlat/nonloc	broad L MFG vs. SFG	Incomplete L HC inversion	L CMT ^4^; LF	Y; Y; 57	N; N/A	100%; 13
19	Fe, R, 15, 28	N; N/A; N/A	N; N/A; N/A	Y; Y, 2	14	U	F; RP	broad R precuneus, RP, R angular gyrus	Unremarkable	R pulvinar ^6^; RP **	Y; Y; 9	N; N/A	100%; 4
20	Fe, R, 14, 33	Y; LF lobectomy; remote infarct	N; N/A; N/A	Y; N, N/A	5	S	F; L FT	broad peri-lesional L MFG/SFG	LF T2 hyperintensity	L ANT ^2,^***; LF	Y; Y; 13	N; N/A	90%; 7
21	Fe, R, 24, 56	N; N/A; N/A	N; N/A; N/A	Y; N, N/A	75	I	F; LT	MF; LT peri-lesion, LT neocortex, L SMG	LT encephalomalacia	L ANT ^2^ then R CMT ^4,^****; LT	N; Y; 44	N; N/A	40%; 16

ANT = anterior nucleus of thalamus, ant = anterior, ATL = anterior temporal lobectomy, Bi = bilateral, BiF = bilateral frontal, BiFT = bilateral frontotemporal, BiP = bilateral parietal, BiPF = bilateral parietofrontal, BiO = bilateral occipital, CMT = centromedian nucleus of thalamus, cort = cortical, Fe = female, F = focal, FCD = focal cortical dysplasia, FT = fronto-temporal, G = generalized, Gen = genetic, HC = hippocampus, I = infectious, ITG = inferior temporal gyrus, L = left, LF = left frontal, LITT = laser interstitial thermal therapy, LT = left temporal, M = male, MCD = malformation of cortical development, MF = multifocal, MFG = middle frontal gyrus, mo = month(s), MV = microvascular, N = no, *n* = number, N/A = not applicable, nonlat = non-lateralizing, nonloc = non-localizing, OFC = orbitofrontal cortex, OSH = outside hospital, PFA = paroxysmal fast activity, R = right, RF = right frontal, RP = right parietal, RT = right temporal, SFG = superior frontal gyrus, SMG = supramarginal gyrus, subcort = subcortical, S = structural, STG = superior temporal gyrus, SANTE = Stimulation of the Anterior Nucleus of the Thalamus for Epilepsy, SEEG = stereoelectroencephalography, TLE = temporal lobe epilepsy, TP = temporoparietal, U = unknown, VNS = vagus nerve stimulator, Y = yes, yr = year, yrs = years. * RNS targets selection: ^1^ Early case implanted before the centromedian nucleus of thalamus became a standard RNS target; ^2^ ANT target for TLE based on SANTE trial results, and mesiotemporal lobe epilepsy or epilepsies involving limbic circuits; ^3^ CMT target for generalized epilepsy; ^4^ CMT target for frontal onset focal epilepsy; ^5^ CMT target for diffuse onset regional epilepsy; ^6^ Pulvinar nucleus target for posterior quadrant epilepsy. ** Patient had right parietal resection post-SEEG and right parietal and right pulvinar RNS lead placement as one procedure. *** L ANT as a thalamic target was selected based on presence of the L FT scalp EEG ictal onset and LT sharp waves and PFA after left frontal cortex resection. **** L ANT as a thalamic target was initially selected based on presence of multifocal left temporal lobe seizures, replaced to CMT based on ineffectiveness and patient-reported seizures suggesting right frontal lobe ictal onset based on seizure semiology which were not detected with L ANT and LT RNS leads.

**Table 2:** Bilateral thalamic and corticothalamic responders’ and nonresponders’ characteristics.

	Bilateral Thalamic Responders (*n* =12)	Bilateral Thalamic Nonresponders (*n* = 5)	Corticothalamic Responders (*n* = 3)	Corticothalamic Nonresponders (*n* = 1)
Median age at epilepsy onset, y (Q1–Q3)	6 (2–10)	5 (2–7)	15 (15–16)	24
Median age at RNS, y (Q1–Q3)	29 (24–44)	23 (19–27)	33 (31–40)	56
Median latency from DRE onset to RNS placement, y (Q1–Q3)	22 (16–39)	20 (18–21)	19 (16–25)	42
Identified Etiology, *n* (%)				
Unknown	9 (75.0%)	3 (60.0%)	1 (33.3%)	0 (0%)
Structural	3 (25.0%)	0 (0.0%)	2 (66.7.3%)	0 (0%)
Genetic	0 (0.0%)	1 (20.0%)	0 (0.0%)	0 (0%)
Infectious	0 (0.0%)	1 (20.0%)	0 (0.0%)	1 (100%)
Median pre-RNS seizure frequency per month (Q1–Q3)	13 (6–45)	30 (30–75)	8 (7–11)	75
Patients who had FBTC/GTC pre-RNS, *n* (%)	9 (75.0%)	2 (40.0%)	3 (100.0%)	1 (100%)
Median pre-RNS FBTC frequency per year (Q1–Q3)	6 (4–28)	125	4	0.5
**Brain MRI**				
Total lesional, *n* (%)	6 (50.0%)	1 (20.0%)	1 (33.3%)	1 (100%)
Lesion(s) related to SEEG ictal onset(s), *n* (%)	2 (16.7%)	1 (100%)	1 (100%)	1 (100%)
Non-lesional, *n* (%)	6 (50.0%)	4 (80.0%)	2 (66.7%)	0 (0.0%)
**Previous neurosurgery**				
Total, *n* (%)	9 (75.0%)	4 (80.0%)	1 (33.3%)	0 (0.0%)
Resection/LITT, *n* (%)	2 (22.2%)	1 (25.0%)	1 (33.3%)	0 (0.0%)
VNS, *n* (%)	5 (55.6%)	2 (50.0%)	0 (0.0%)	0 (0.0%)
Resection/LITT + VNS, *n* (%)	2 (22.2%)	1 (25.0%)	0 (0.0%)	0 (0.0%)
**SEEG Ictal Onset**				
Not completed	4 (33.3%)	2 (40.0%)	0 (0.0%)	0 (0.0%)
Broad	3 (25%)	1 (20.0%)	3 (100.0%)	0 (0.0%)
Multifocal	5 (41.7%)	2 (40.0%)	0 (0.0%)	1 (100%)
**RNS**				
CMT targets, *n* (%)	7 (58.3%)	4 (80%)	1 (33.3%)	1 (100%)
ANT targets, *n* (%)	5 (41.7%)	1 (20%)	1 (33.3%)	0 (0.0%)
Pulvinar targets, *n* (%)	0 (0%)	0 (0%)	1 (33.3%)	0 (0.0%)
Median follow-up duration, m (Q1–Q3)	35 (23–51)	47 (15–60)	13 (11–35)	44
Median percentage reduction in seizures (Q1–Q3)	75% (74–81)	30% (10–40)	100% (95–100)	40%
Patients who had FBTC/GTC at last follow-up, *n* (%)	4 (33.3%)	1 (20.0%)	0 (0.0%)	0 (0.0%)
Median FBTC/GTC frequency at last follow-up per year (Q1–Q3)	1 (1–8)	125	0	0
Patients who had reduction in seizure severity	11 (91.7%)	4 (80.0%)	3 (100%)	1 (100%)

ANT = anterior nucleus of thalamus, CMT = centromedian nucleus of thalamus, FBTC = focal to bilateral tonic–clonic, GTC = generalized tonic–clonic, LITT = laser interstitial thermal therapy, m = month, *n* = number, (Q1–Q3) = quartile 1 to quartile 3, RNS = responsive neurostimulation, SEEG = stereoelectroencephalography, VNS = vagal nerve stimulator, y = year.

**Table 3:** RNS therapy outcomes at common intervals.

Patient ID	Thalamic Nucleus Target; Cortical Target (If Placed)	≥50% Seizure Reduction (Y/N); Seizure Severity Reduction (Y/N)							Follow-Up Duration (Months)
		6 mo f/u	1 y f/u	2 y f/u	3 y f/u	4 y f/u	5 y f/u	Recent f/u	
Bilateral thalamic leads									
1	Bi CMT	Y; N	Y; N	Y; N	Y; Y	Y; Y	Y; Y	Y; Y	67
2	Bi CMT	N; N	Y; N	Y; N	Y; Y	Y; Y	N/A	Y;Y	49
3	Bi CMT	N; Y	N; N	Y; N	N/A	N/A	N/A	Y; N	29
4	Bi CMT	N; Y	Y; Y	Y; Y	N/A	N/A	N/A	Y; Y	25
5	Bi CMT	Y; N	Y; N	Y; Y	N/A	N/A	N/A	Y; Y	21
6	Bi CMT	N; N	Y; Y	N/A	N/A	N/A	N/A	Y; Y	15
7	Bi CMT	Y; N	Y; Y	N/A	N/A	N/A	N/A	Y; Y	14
8	Bi ANT	N; Y	Y; Y	Y; Y	Y; Y	Y; Y	Y; Y	Y; Y	76
9	Bi ANT	Y; Y	Y; Y	Y; Y	Y; Y	Y; Y	N/A	Y; Y	55
10	Bi ANT	N; N	Y; Y	Y; Y	Y; Y	Y; Y	N/A	Y; Y	49
11	Bi ANT	N; Y	N; Y	N; Y	Y; Y	N/A	N/A	Y; Y	41
12	Bi ANT > Bi HC	N; N	N/A	Y; N	Y; Y	N/A	N/A	Y; Y	23
13	Bi CMT	N; N	N; N	N; Y	N; Y	N/A	N/A	N; Y	47
14	Bi CMT	N; N	N; N	N/A	N/A	N/A	N/A	N; N	15
15	Bi CMT	N; N	N; Y	N/A	N/A	N/A	N/A	N; Y	15
16	Bi ANT > Bi CMT	N; Y	N; Y	N; Y	N; Y	N; Y	N; Y	N; Y	81
17	Bi ANT	N; Y	N; Y	N; Y	N; Y	N; Y	N; Y	N; Y	60
Corticothalamic leads									
18	L CMT; LF	N/A	Y; Y	Y; Y	Y; Y	Y; Y	N/A	Y; Y	57
19	R pulvinar; RP	Y; Y	Y; Y	N/A	N/A	N/A	N/A	Y; Y	9
20	L ANT; LF	Y; Y	Y; Y	N/A	N/A	N/A	N/A	Y; Y	13
21	L ANT > R CMT; LT	N; N	N; N	N; N	N; Y	N/A	N/A	N; Y	44

ANT = anterior nucleus of thalamus, Bi = bilateral, CMT = centromedian nucleus of thalamus, f/u = follow-up, HC = hippocampus, L = left, LF = left frontal, LT = left temporal, mo = months, N = no, R = right, RP = right parietal, Y = yes, y = years.

**Table 4:** Anti-seizure medications pre-RNS and at the most recent RNS follow-up visit.

Patient ID	Thalamic Nucleus Target; Cortical Target (If Placed)	Number of ASMs Pre-RNS//at Last RNS f/u	Pre-RNS ASM Regimen	Most Recent Follow-Up Post-RNS ASM Regimen
Bilateral thalamic leads				
1	Bi CMT	4//3	BRV 200 mg BID, CBD 420 mg BID, TPM 400 mg qhs, PHT ER 200 mg BID	BRV 200 mg BID, CNB 200 mg qhs, TPM 400 mg qhs
2	Bi CMT	2//4	BRV 100 mg BID, CLB 20 mg BID	CBD 1000 mg BID, BRV 150 mg BID, CLB 20 mg BID, ZNS 200 mg qhs
3	Bi CMT	2//1	LTG 450/375 mg BID, Perampanel 6 mg qhs	LTG 450 mg BID
4	Bi CMT	2//2	FBM 1200 mg BID, LTG 100 mg BID	FBM 1200 mg BID, LTG 150 mg BID
5	Bi CMT	2//3	LEV 1000/1500 mg BID, OXC 1500 mg BID	LEV 1500 mg BID, OXC 1500 mg BID, CNB 200 mg qhs
6	Bi CMT	3//3	CLB 20 mg qhs, OXC 300 mg BID, PHT 100 mg TID	CLB 20 mg TID, OXC 300 mg BID, PHT 300 mg TID
7	Bi CMT	1//1	CNB 200 mg qhs	CNB 200 mg qhs
8	Bi ANT	3//3	BRV 100 mg BID, CBZ 400 mg BID, ZNS 700 mg qhs	BRV 100 mg BID, CBZ 200 mg BID, ZNS 200 mg qhs
9	Bi ANT	3//4	CBZ 400 mg BID, LCM 525 mg BID, LEV 3000 mg BID	CBZ 200 mg BID, CLB 30 mg qhs, LCM 525 mg BID, LEV 3000 mg BID
10	Bi ANT	2//2	BRV 200 mg BID, LTG 300 mg BID	BRV 200 mg BID, LTG 300 mg BID
11	Bi ANT	3//3	CBZ 600 mg BID, LEV 750 mg BID, ZNS 500 mg qhs	CBZ 400 mg BID, LEV 750/1500 mg, ZNS 200/300 mg
12	Bi ANT > Bi HC	4//4	TPM 150/150/100 mg, LCM 200 mg TID, CLB 5/15 mg BID, Lyrica 300 mg qhs	TPM 200/200/100 mg, LCM 200 mg TID, CLB 5/20 mg BID, Lyrica 100 mg TID
13	Bi CMT	3//3	FBM 1800/1200 mg BID, CLB 20 mg BID, VPA 500 mg BID	FBM 800/1600 mg BID, CLB 5 mg BID, CNB 325 mg qhs
14	Bi CMT	3//3	CNB 200 mg qhs, FBM 600 mg TID, OXC 600 mg BID	CNB 400 mg qhs, FBM 900 mg BID, OXC 600 mg BID
15	Bi CMT	2//4	LCM 200 mg BID, TPM 200 mg BID	BRV 100 mg BID, CNB 300 mg qhs, LCM 250 mg BID, TPM 200 mg BID
16	Bi ANT > Bi CMT	4//3	CNB 450 mg qhs, CLB 20 mg qhs, LCM 400 mg BID, TPM 200 mg BID	CLB 10 mg BID, LCM 400 mg BID, TPM 600 mg qd
17	Bi ANT	2//2	VPA 1000 mg qhs, LTG 150 mg BID	LCM 100 mg BID, LTG 150 mg BID
Corticothalamic leads				
18	L CMT; LF	2//3	ESL 800 mg BID, ZNS 100/200 mg BID	ESL 400/800 mg BID, CNB 150 mg BID, ZNS 100 mg qhs
19	R pulvinar; RP	3//3	LTG 300 mg BID, ZNS 200 mg BID, CLB 20 mg BID	LTG 400 mg BID, ZSM 500 mg daily, CLB 20 mg BID
20	L ANT; LF	3//2	BRV 100 mg BID, OXC 1200 mg BID, CLB 10/20 mg BID	BRV 200 mg BID, CNB 400 mg qhs
21	L ANT > R CMT; LT	2//2	LSM 50/100/100 mg TID, LEV 2000 mg BID	LSM 100/200 mg BID, LEV 1500/2000 mg BID

ANT = anterior nucleus of thalamus, ASM = anti-seizure medications, Bi = bilateral, BID = twice a day, BRV = brivaracetam, CBD = cannabidiol, CLB = clobazam, CNB = cenobamate, CMT = centromedian nucleus of thalamus, ER = extended release, ESL = eslicarbazepine, FBM = felbamate, f/u = follow-up, L = left; LCM = lacosamide, LEV = levetiracetam, LF = left frontal, LT = left temporal, LTG = lamotrigine, mg = milligrams, OXC = oxcarbazepine, PHT = phenytoin, qhs = “quaque hora somni” meaning “every night at bedtime”, R = right, RNS = responsive neurostimulation, RP = right parietal, TID = three times a day, TPM = topiramate, VPA = valproic acid, ZNS = zonisamide.

## Data Availability

The original contributions presented in this study are included in the article. Further inquiries can be directed to the corresponding author.

## Supplementary Materials

The following supporting information can be downloaded at https://www.mdpi.com/article/10.3390/neurosci7040092/s1.

## References

[1] Perucca E., Perucca P., White H.S., Wirrell E.C. (2023). Drug resistance in epilepsy. Lancet Neurol..

[2] Heck C.N., King-Stephens D., Massey A.D., Nair D.R., Jobst B.C., Barkley G.L., Salanova V., Cole A.J., Smith M.C., Gwinn R.P. (2014). Two-year seizure reduction in adults with medically intractable partial onset epilepsy treated with responsive neurostimulation: Final results of the RNS System Pivotal trial. Epilepsia.

[3] Eliashiv D., Rao V.R., Jobst B.C., Szaflarski J.P., Rolston J.D., Kaye L.C., Ganguly T.M., Bullinger K., Dugan P.C., Burdette D.E. (2026). Postapproval Study for Brain-Responsive Neurostimulation for Drug-Resistant Focal Epilepsy: Three-Year Efficacy and Interim Safety Results. Neurology.

[4] Velasco F., Velasco M., Ogarrio C., Fanghanel G. (1987). Electrical Stimulation of the Centromedian Thalamic Nucleus in the Treatment of Convulsive Seizures: A Preliminary Report. Epilepsia.

[5] Manjunatha R.T., Vakilna Y.S., Chaitanya G., Alamoudi O., Ilyas A., Pati S. (2023). Advancing the frontiers of thalamic neuromodulation: A review of emerging targets and paradigms. Epilepsy Res..

[6] Beaudreault C.P., Muh C.R., Naftchi A., Spirollari E., Das A., Vazquez S., Sukul V.V., Overby P.J., Tobias M.E., McGoldrick P.E. (2022). Responsive Neurostimulation Targeting the Anterior, Centromedian and Pulvinar Thalamic Nuclei and the Detection of Electrographic Seizures in Pediatric and Young Adult Patients. Front. Hum. Neurosci..

[7] Fisher R., Salanova V., Witt T., Worth R., Henry T., Gross R., Oommen K., Osorio I., Nazzaro J., Labar D. (2010). Electrical stimulation of the anterior nucleus of thalamus for treatment of refractory epilepsy. Epilepsia.

[8] Feigen C.M., Eskandar E.N. (2022). Responsive Thalamic Neurostimulation: A Systematic Review of a Promising Approach for Refractory Epilepsy. Front. Hum. Neurosci..

[9] Hsu J., Ali R., Air E., Englot D.J., Muh C.R., Richardson R.M., Rammo R. (2026). Thalamic Neuromodulation: New Frontiers. Epilepsy Curr..

[10] Burdette D., Patra S., Johnson L. (2024). Corticothalamic Responsive Neurostimulation for Focal Epilepsy: A Single-Center Experience. J. Clin. Neurophysiol..

[11] Agashe S., Alcala-Zermeno J.L., Osman G.M., Starnes K., Sheffield W.D., Leyde K., Stead M., Brinkmann B.H., Miller K.J., Van Gompel J.J. (2025). Thalamocortical network neuromodulation for epilepsy. Brain Commun..

[12] Khambhati A.N., Shafi A., Rao V.R., Chang E.F. (2021). Long-term brain network reorganization predicts responsive neurostimulation outcomes for focal epilepsy. Sci. Transl. Med..

[13] Yu T., Wang X., Li Y., Zhang G., Worrell G., Chauvel P., Ni D., Qiao L., Liu C., Li L. (2018). High-frequency stimulation of anterior nucleus of thalamus desynchronizes epileptic network in humans. Brain.

[14] Roa J.A., Abramova M., Fields M., La Vega-Talbott M., Yoo J., Marcuse L., Wolf S., McGoldrick P., Ghatan S., Panov F. (2022). Responsive Neurostimulation of the Thalamus for the Treatment of Refractory Epilepsy. Front. Hum. Neurosci..

[15] Fields M.C., Eka O., Schreckinger C., Dugan P., Asaad W.F., Blum A.S., Bullinger K., Willie J.T., Burdette D.E., Anderson C. (2023). A multicenter retrospective study of patients treated in the thalamus with responsive neurostimulation. Front. Neurol..

[16] Burdette D.E., Haykal M.A., Jarosiewicz B., Fabris R.R., Heredia G., Elisevich K., Patra S.E. (2020). Brain-responsive corticothalamic stimulation in the centromedian nucleus for the treatment of regional neocortical epilepsy. Epilepsy Behav..

[17] Elder C., Friedman D., Devinsky O., Doyle W., Dugan P. (2018). Responsive neurostimulation targeting the anterior nucleus of the thalamus in 3 patients with treatment-resistant multifocal epilepsy. Epilepsia Open.

[18] Uysal U., Landazuri P., Burdette D.E., Patra S., Crudele A.N., Englot D.J., Gavvala J.R., Pati S., Kaye L.C., Ojemann S. (2026). Responsive stimulation of the thalamus for idiopathic generalized epilepsy: Results of the randomized controlled NAUTILUS trial through 18 months. Epilepsia.

[19] Cukiert A., Cukiert C.M., Burattini J.A., Mariani P.P. (2020). Seizure outcome during bilateral, continuous, thalamic centromedian nuclei deep brain stimulation in patients with generalized epilepsy: A prospective, open-label study. Seizure.

[20] Wong G.M., Hofmann K., Shlobin N.A., Tsuchida T.N., Gaillard W.D., Oluigbo C.O. (2023). Stimulation of the pulvinar nucleus of the thalamus in epilepsy: A systematic review and individual patient data (IPD) analysis. Clin. Neurol. Neurosurg..

[21] Burdette D., Mirro E.A., Lawrence M., Patra S.E. (2021). Brain-responsive corticothalamic stimulation in the pulvinar nucleus for the treatment of regional neocortical epilepsy: A case series. Epilepsia Open.

[22] Hu T., Xie H., Shan M., Sang L., Yang B., Zhang Q., Gao D., Jiao Y., Guo Q., Li S. (2025). Long-term efficacy of anterior nucleus of the thalamus deep brain stimulation in drug-resistant epilepsy: A 5-year multicenter study. BMC Med..

[23] Ryvlin P., Rheims S., Hirsch L.J., Sokolov A., Jehi L. (2021). Neuromodulation in epilepsy: State-of-the-art approved therapies. Lancet Neurol..

[24] Mabry M.H., Podkorytova I., Chinedu-Eneh E., Alick-Lindstrom S., Ding K., Hays R., Perven G. (2026). Responsive Neurostimulation in Patients with a History of Viral Brain Infections—A Single-Center Experience. NeuroSci.

[25] Subramaniam V.R., Chan A.H.W., Marcuse L., Fields M., La Vega-Talbott M., Cummins D.D., Barcia J.A., Ghonim H.T., Jayagopal L.A., Im Y. (2026). Connectivity between the seizure onset zone and the thalamus correlates with seizure outcomes in thalamic responsive neurostimulation. Epilepsia.

[26] Jo H.J., Kenny-Jung D.L., Balzekas I., Benarroch E.E., Jones D.T., Brinkmann B.H., Stead S.M., Van Gompel J.J., Welker K.M., Worrell G.A. (2019). Nuclei-specific thalamic connectivity predicts seizure frequency in drug-resistant medial temporal lobe epilepsy. NeuroImage Clin..

[27] Middlebrooks E.H., He X., Grewal S.S., Keller S.S. (2022). Neuroimaging and thalamic connectomics in epilepsy neuromodulation. Epilepsy Res..

[28] Jobst B.C., Kapur R., Barkley G.L., Bazil C.W., Berg M.J., Bergey G.K., Boggs J.G., Cash S.S., Cole A.J., Duchowny M.S. (2017). Brain-responsive neurostimulation in patients with medically intractable seizures arising from eloquent and other neocortical areas. Epilepsia.

[29] Wu T.Q., Kaboodvand N., McGinn R.J., Veit M., Davey Z., Datta A., Graber K.D., Meador K.J., Fisher R., Buch V. (2023). Multisite thalamic recordings to characterize seizure propagation in the human brain. Brain.

[30] Feys O., Pizzo F., Makhalova J., Carron R., Bartolomei F. (2025). The role of the thalamus in focal human epilepsy: Insights from stereoelectroencephalography (SEEG). Front. Neurol..

[31] Nair D.R., Laxer K.D., Weber P.B., Murro A.M., Park Y.D., Barkley G.L., Smith B.J., Gwinn R.P., Doherty M.J., Noe K.H. (2020). Nine-year prospective efficacy and safety of brain-responsive neurostimulation for focal epilepsy. Neurology.

[32] Kanai S., Daida A., Zhang Y., Ding Y., Monsoor T., Qiao J.X., Salamon N., Ronne E., Ahn S.S., Hussain S.A. (2025). Optimizing responsive neurostimulation targeting based on interictal high-frequency oscillations and phase–amplitude coupling. Epilepsia.

[33] Sheybani L., Qiu Y., Singh P.C., Vivekananda U., Burgess N., Diehl B., McEvoy A., Miserocchi A., Bisby J.A., Shekh-Ahmad T. (2026). Fast-ripples are emergent properties of neuronal networks. BioRxiv.

