# Supplementary material for: Responsive Neurostimulation Therapy Outcomes in Two Small Bilateral Thalamic and Corticothalamic Subgroups of Patients with Drug-Resistant Epilepsy—A Single-Center Experience
Source: NeuroSci. 2026 Aug 21;7(4):92. doi: 10.3390/neurosci7040092 (PMC13516396; doi:10.3390/neurosci7040092)
Supplement: Supplementary file 1 [file neurosci-07-00092-s001.zip › Figure S1.pdf]

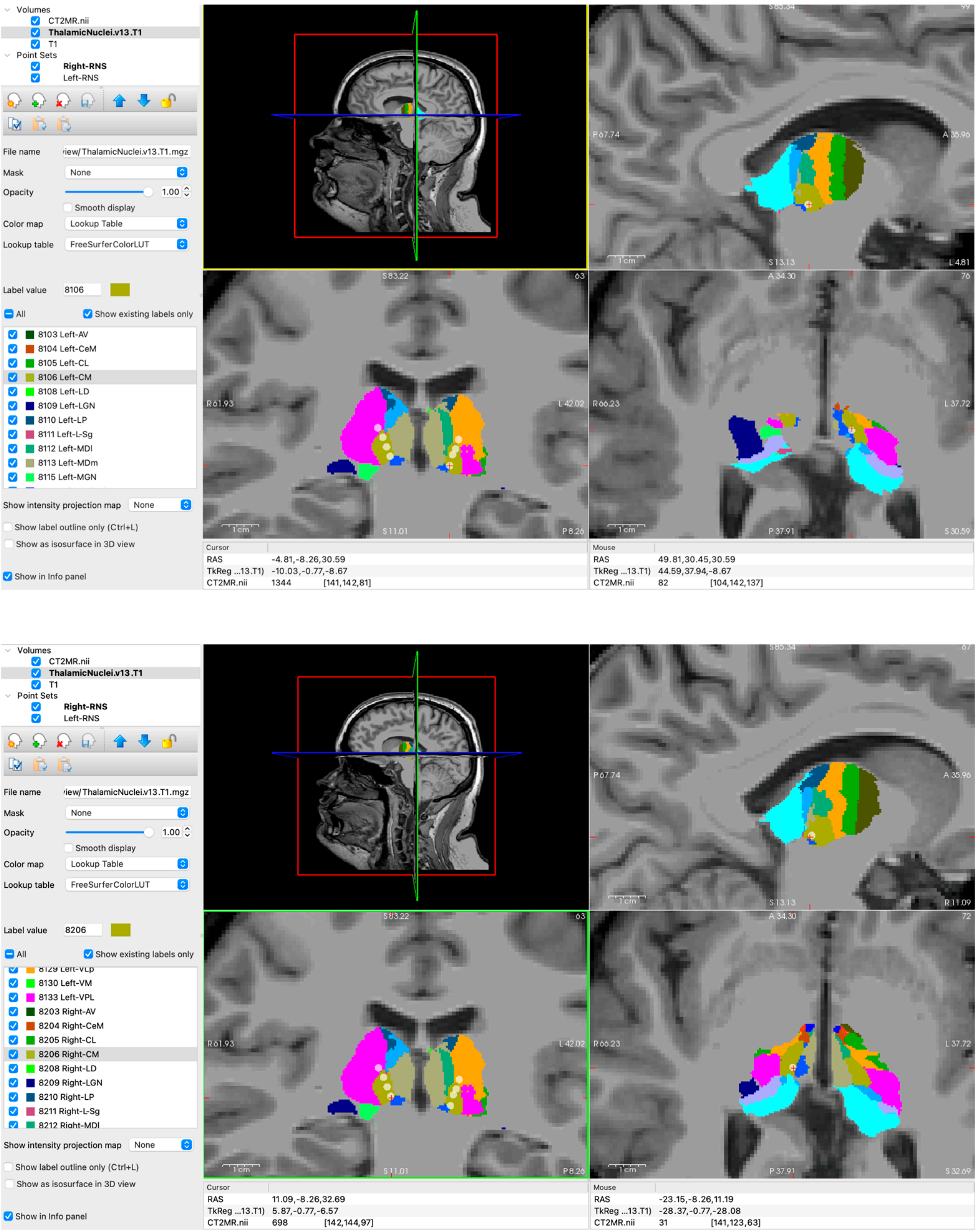

Figure S1. Patient 15, the RNS leads location in the centromedian nucleus of thalamus bilaterally seen in Freesurfer software. Top, left RNS lead; bottom, right RNS lead.
